# Common Features in lncRNA Annotation and Classification: A Survey

**DOI:** 10.3390/ncrna7040077

**Published:** 2021-12-13

**Authors:** Christopher Klapproth, Rituparno Sen, Peter F. Stadler, Sven Findeiß, Jörg Fallmann

**Affiliations:** 1 Bioinformatics Group, Department of Computer Science, and Interdisciplinary Center for Bioinformatics, University of Leipzig, Härtelstraße 16-18, D-04107 Leipzig, Germany; christopher@bioinf.uni-leipzig.de (C.K.); studla@bioinf.uni-leipzig.de (P.F.S.); sven@bioinf.uni-leipzig.de (S.F.); 2 Helmholtz Institute for RNA-Based Infection Research (HIRI), Helmholtz-Center for Infection Research (HZI), D-97080 Würzburg, Germany; rituparno@bioinf.uni-leipzig.de; 3 German Centre for Integrative Biodiversity Research (iDiv) Halle-Jena-Leipzig, Competence Center for Scalable Data Services and Solutions, and Leipzig Research Center for Civilization Diseases, University Leipzig, D-04103 Leipzig, Germany; 4 Max Planck Institute for Mathematics in the Sciences, Inselstraße 22, D-04103 Leipzig, Germany; 5 Institute for Theoretical Chemistry, University of Vienna, Währingerstraße 17, A-1090 Vienna, Austria; 6 Facultad de Ciencias, Universidad National de Colombia, Bogotá CO-111321, Colombia; 7 Santa Fe Institute, 1399 Hyde Park Rd., Santa Fe, NM 87501, USA

**Keywords:** lncRNA, feature extraction, machine learning, coding sequence, classification problems

## Abstract

Long non-coding RNAs (lncRNAs) are widely recognized as important regulators of gene expression. Their molecular functions range from miRNA sponging to chromatin-associated mechanisms, leading to effects in disease progression and establishing them as diagnostic and therapeutic targets. Still, only a few representatives of this diverse class of RNAs are well studied, while the vast majority is poorly described beyond the existence of their transcripts. In this review we survey common in silico approaches for lncRNA annotation. We focus on the well-established sets of features used for classification and discuss their specific advantages and weaknesses. While the available tools perform very well for the task of distinguishing coding sequence from other RNAs, we find that current methods are not well suited to distinguish lncRNAs or parts thereof from other non-protein-coding input sequences. We conclude that the distinction of lncRNAs from intronic sequences and untranslated regions of coding mRNAs remains a pressing research gap.

## 1. Introduction

A substantial fraction of transcribed RNA does not code for proteins. Over the last decade, it has become evident that many of these non-coding transcripts play an important role as regulators of gene expression [1,2,3,4]. Especially the subclass of long non-coding RNAs (lncRNAs) is also associated with a wide array of diseases, in particular cancer, and hence have gained importance in medical research [5,6,7,8]. The identification and annotation of lncRNAs thus has become a problem of considerable practical importance. Nevertheless, only a minimal fraction of the lncRNAs catalogued so far are also associated with functional annotation.

Machine learning (ML) has become the de facto standard for automated annotation of lncRNAs. The advent of third generation sequencing techniques and reinforced efforts in genome annotation have also led to rapid increase in the amount and quality of data on lncRNAs, enabling significant breakthroughs in the field [9,10]. Still, the distinction of protein-coding transcripts and lncRNAs has remained a non-trivial problem, in particular since lncRNAs and protein-coding mRNAs share features such as length and genomic organization into introns and exons [11].

Here we survey the broad range of currently available computational tools for lncRNA annotation and function prediction with a focus on the features that are used in the decision processes. These range from straightforward sequence-based parameters (such as nucleotide or *k*-mer frequencies) to predictions of complex physico-chemical properties of a hypothetically encoded protein. There are many commonalities among the methods reviewed below. In essence, many tools compute parameters that quantify coding potential [12], and recently developed alternative descriptors are in many cases only slight variations on this theme.

## 2. Commonly Used Features in Non-Coding Transcript Annotation

Most classical machine learning approaches that address lncRNAs are built around limited sets of features owing to the limited catalogues of well-annotated lncRNAs that were available until very recently. Naturally, these features are designed to capture the “coding potential” of transcripts or subsequences of interest. These approaches are obviously based on the hypothesis that there is clear-cut and measurable distinction between coding and non-coding transcripts. The existence of very short translated open reading frames (ORFs) [13,14,15] and increasing evidence for genetic regions that produce isoforms with both coding and a non-coding mode of action [16,17], however, blurs the distinction.

**Figure 1:**
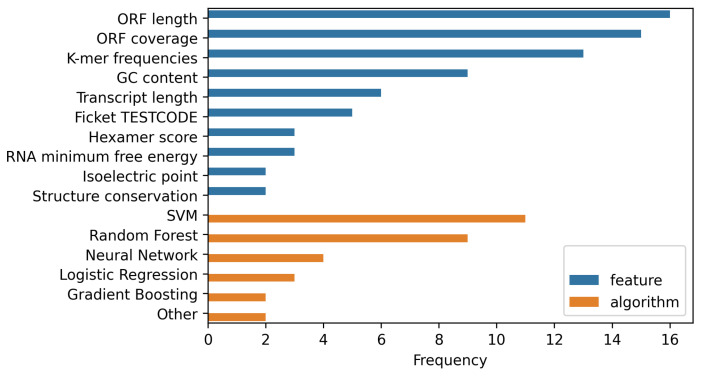
A comparison of the frequencies of commonly used features (blue) and algorithms (orange) applied by different contemporary tools. A majority of the latter rely on open reading frame (ORF) information to make predictions. Other often utilized features include subsequence (*k*-mer) frequencies and GC content. SVMs and Random Forests dominate the field as the most commonly implemented algorithms. This is not surprising, as they are two of the by far most flexible approaches for nonlinear classification.

Well-established features and algorithms for lncRNA classification are summarized in Figure 1 and the following section.

- *k*-mers are *k*-letter subsequences of a given sequence stretch. A single base is a 1-mer, whereas codons are base-triplets and thus 3-mers. The information encoded by *k*-mers is distributed based on the value of *k*. Shorter *k*-mers are more abundant and their relative frequencies are more strongly cross-correlated than for longer *k*-mers. A  7-mer would encode more information than a 3-mer, for example, as the probability of occurrence of a particular 7-mer is much lower than that of a 3-mer. However, frequencies of longer *k*-mers become computationally more expensive to calculate [18,19]: For k=7 there are already 16,384 distinct features, which translates to an at least equal number of subsequent comparison operations. In addition to the computational issues, sufficiently large training sets and test sets are required.
- *Euclidean and logarithmic distances* of the frequency vectors of certain features such as *k*-mer frequencies relative to the expected values of these frequencies in a reference set of lncRNAs and protein-coding sequences, respectively, are utilized in, e.g., LncFinder [20].
- *GC content* is the number of purine bases (either G or C) in the sequence normalized by the length of the sequence. Higher GC content tends to be closely associated with the presence of RNA secondary structure which in turn tends to correlate with biological function aside from the encoding of proteins [21]. The GC content can therefore serve as an indicator for coding potential.
- *Fickett TESTCODE* was the first method proposed to find a distinguishing factor between the two classes of RNA [22]. To circumvent the hard problem of identifying initiation signals in a sequence, the authors devised a test to identify whether a given piece of DNA or RNA is coding or non-coding directly from the sequence. It is based on the asymmetric distribution of bases in protein-coding sequences. The test is based on eight different parameters, the first four being measures of the bases A, T, G and C, calculating the probabilities for them being favored in one of the three codon positions. The latter four are the ratios of each base in the sequence. Attaching weights to each parameter, the coding potential is computed, called TESTCODE or Fickett score.
  (1) $$Fickett\;score=\sum_{i=1}^{8}p_{i}w_{i}.$$
  The detection tool CPAT [23], derives the probability of a base B∈{A,C,G,T} being favored at a certain position as:
  $$\begin{array}{cc} B_{1} & =\mathrm{Cardinality}\;\mathrm{of}\;\mathrm{base}\;B\;\mathrm{in}\;\mathrm{position}\;0,\;3,\;6,\;\ldots \\ B_{2} & =\mathrm{Cardinality}\;\mathrm{of}\;\mathrm{base}\;B\;\mathrm{in}\;\mathrm{position}\;1,\;4,\;7,\;\ldots \\ B_{3} & =\mathrm{Cardinality}\;\mathrm{of}\;\mathrm{base}\;B\;\mathrm{in}\;\mathrm{position}\;2,\;5,\;8,\;\ldots \\ B_{pos} & =\frac{\max(B_{1},B_{2},B_{3})}{\min(B_{1},B_{2},B_{3})+1}. \end{array}$$
  The derived values are then converted into probabilities (*p*) using the lookup table provided by Fickett [22] or an updated table taking into account newly annotated transcripts, as provided for example by Wang et al. [23]. In principle, this gives a measure for how non-random a nucleotide is distributed across 3-mers of a given sequence. Furthermore, *w* in Equation (1) is a weight representing the predictive power of each parameter on its own given sequences with an already known function. It has been shown that the Ficket score can achieve 94% and 97% sensitivity and specificity, respectively, on lncRNA sequences, being inconclusive for 18% of the sequences [23].
- *Hexamer score.* Adjacent amino acids in a protein are not independent [23], especially with regards to their direct neighbors. This results in a hexamer usage bias that can be used to calculate a relative score. Coding sequences usually derive a positive, non-coding sequences a negative score [24]. There are several ways of defining a hexamer score. The strategy used in CPAT [23] computes the log-likelihood ratio between coding and non-coding sequences. The score for a sequence S = $H_{1},H_{2},\ldots ,H_{m}$ with *m* hexamers is derived as:
  $$hexamer\_score=\frac{1}{m}\sum_{i=1}^{m}\log\left(\frac{F(H_{i})}{F^{\prime }\left(H_{i}\right)}\right),$$
  where $F(H_{i})$ and $F^{\prime }\left(H_{i}\right)$ represent the probability of each hexamer to be part of a protein-coding and non-coding sequence, respectively, with  4096 total hexamers possible.
- *ORF length.* Translation of mRNAs into proteins by the ribosome begins at a start codon (AUG) and ends at one of the stop codons (UAA/UGA/UAG) of spliced mRNA. Thus, ORFs are the theoretically translatable subunits of genes and therefore of functional relevance. The length of a putative ORF is a feature frequently used to predict if a sequence has any coding potential [12]. It should be noted that not every ORF translatable on a sequence level is in fact translated to a peptide chain.
- *ORF coverage.* The ratio between the ORF length and the length of the sequence in consideration is used. Long ORFs are generally considered as an indicator of a coding sequence. Following that logic, a low coverage indicates a non-coding sequence [25].
- *Peptide length.* The hypothetically translated amino acid (AA) sequence of a given ORF can be analyzed for physico-chemical and general properties. The length of this AA-sequence corresponds to the peptide length. Note that this property is obviously linked to the  ORF length.
- *Hydropathy* is a measure of hydrophilic or hydrophobic interactions of a potential peptide sequence with a water-based solvent. Since amino acids are categorized as either hydrophobic, hydrophilic or neutral, this property also implicitly contains information regarding the AA composition of a protein. A hydropathic index is typically calculated as a contribution function from all individual amino acid residues present in a peptide chain, see [26] for a review of amino acid hydrophobicity scales.
- *Isoelectric point:* The *isoelectric point* (pI) refers to the pH value where a balance between negative and positive charges in an amphoteric molecule is reached. As practically all relevant proteins are amphoteric, the pI is a relevant measure for the AA composition. Different residues contribute different physico-chemical properties depending on their free electron pairs. As with the hydropathic score the pI is usually approximated using the contribution of all present residues in a given peptide sequence, see, e.g., [27].
- *PolyA abundance:* The presence of polyadenylation signals in a transcript is a strong indicator for a protein coding function. Polyadenylation typically happens at the 3′-terminus of mature mRNAs in eukaryotic cells. It is suggested to increase stability of the transcript while simultaneously regulating its durability. The proportion of PolyA signals (5′-AAUAAA-3′) as a fraction of sequence length can be used a measure of coding potential.
- *RNA minimum free energy* (MFE) is often used as a metric for the inherent stability of the RNA secondary structure of a given nucleotide sequence and refers to the thermodynamic energy of the most stable conformation. Although the MFE does not necessarily correspond to the true RNA secondary structure adopted in vivo it is often used as reasonable approximation. Since non-coding RNAs tend to have a much higher degree of conserved structural complexity, the MFE can be used to infer sequences with a comparably low coding potential.

## 3. Contemporary lncRNA Detection Strategies

Most strategies to distinguish protein-coding and non-coding sequences developed over the last two decades defined the problem as binary classification task. Often a combination of features as described in Section 2 are applied to separate these two classes based on their coding potential [25]. In these efforts, ORF-related features in particular are often utilized, following the idea that these capture information regarding overall viability of a hypothetical protein. Shorter ORF length and lower ORF coverage are often interpreted as signals for non-coding function [28,29]. Another common strategy involves the decomposition of a candidate sequence into *k*-mer patterns and relies on an inherent *k*-mer frequency bias in sequences with coding function. Most tools analyzed for the purpose of this review use a combination of different features to increase combined effectiveness. Furthermore, we limited this list to tools that perform the classification on an individual input sequence without the need for a pairwise or multiple sequence alignment. Alignment-free classifiers have the advantage of being applicable also to species with few or no closely related neighbors with which reliable alignments could be constructed. Since the de novo prediction and annotation of lncRNAs in such species is of great research interest and potential for our understanding of the evolution of non-coding RNAs, we consider tools that facility this to be our primary focus.

Tools focusing on the identification of non-coding and functional RNA elements based on alignments have a long track record of high success in the field of lncRNA detection [30] and beyond. The primary reason for this lies in the ability of such approaches to assess every sequence not only on its own properties but also in its relative evolutionary context, which can reveal conservation patterns that are invisible on the level of the individual transcript [31]. Prime examples that follow this strategy include frameworks such as RNAz [32], cmfinder [33] and PhyloCSF [34,35]. Evolutionary conservation can be assessed on a structural and sequence level, where the conservation of secondary structure serves as an indicator for biological function beyond the encoding of a peptide sequence [36]. Conversely, the conservation of an encoded amino acid sequence where most mutations on the nucleotide level are silent and conserve codons is more closely associated with protein coding transcripts [37,38].

As an approximate measurement of the relative scientific impact of each of the tools discussed here, we included the average citations per year in Table 1. We restricted a more detailed analysis to 22 of the 31 tools that received an average of at least 10 citations per year. For the purpose of categorization, approaches were grouped by type and extent of sequence information measured or inferred for feature extraction. In this regard, tools were grouped into four categories: (3.1) Purely sequence based strategies, that entirely rely on the information contained in sequence composition itself, (3.2) approaches that further assess potential open reading frame properties, (3.3) strategies that also infer properties of hypothetically encoded proteins and (3.4) tools that additionally evaluate properties of (predicted) secondary structure.

### 3.1. Strategies Based on Subsequence Decomposition

**PLEK** [18] is an alignment-free tool designed to distinguish between mRNAs and lncRNAs utilizing Support Vector Machines(SVMs) with a radial basis function kernel. PLEK implements *k*-mers as its core feature. Choosing *k* values from 1 to 5, the authors computed a weight function for each *k*-mer length with a sliding window approach to derive the relationship between each *k*-mer and the sliding window. The SVM is trained as a binary classifier on human protein-coding and long non-coding sequences. The tool achieved more than 95% accuracy on 10-fold cross-validation (CV). Cross-species validation returned similar accuracy levels as the tool Coding-Non-Coding index (CNCI) on same unseen data. Human, mouse and other vertebrate transcripts were collected from Ensembl [39], RefSeq [40] and GENCODE [41].

**DeepLNC** [42] is a tool based on deep neural networks (DNN). The only feature set used here is *k*-mer frequency combinations for *k* values ranging from 2 to 5 with counts normalized by the Shannon entropy. The authors implemented a binary classification model based on DNN to separate mRNA transcripts from lncRNA transcripts. Sample sequences were taken from RefSeq and LNCipedia [43] and limited to human. The tool reached accuracy levels of 98% on 10-fold CV with *k*-mer combinations of 2, 3 and 5. As the method relies on already pre-identified transcripts, it is not suitable for ’blind’ whole genome screens.

**BASiNet** [44] uses a decision tree approach to classify RNA based on features extracted from weighted graph representation. A given sequence is converted to an undirected weighted network of nodes and vertices. Nodes in this representation correspond to substrings of different lengths where the edges and weights indicate their neighborhood in the sequence. Therefore, the final network represents the organization of adjacency of all possible *k*-mers up to a certain length. Topological features of the network are extracted as a feature vector. In the second step, all edges below a certain threshold weight are cut and the same features calculated for the reduced graph. This step is repeated with increasing threshold values until no edges are left and a feature vector is created with every reduction. For the final classification a normalized feature vector is calculated. The method was evaluated with several thousand transcripts from multiple vertebrate and one plant species and achieved an average accuracy of 99.7%. Training and test sets were constructed from RefSeq and GENCODE and EnsemblPlants [45].

### 3.2. Approaches Considering Open Reading Frames (ORF) and Most-Likely Coding Sequences

**iSeeRNA** [46] is another SVM-based identification tool. It leverages three feature categories comprising conservation scores, ORF details and nucleotide sequences. The mean phastCons score [47,48] of every nucleotide for a sequence is calculated together with putative ORF length and coverage. The last feature category constitutes two 2-mers and five 3-mers (GC, CT, TAG, TGT, ACG, TCG) with the fifth 3-mer not mentioned. iSeeRNA uses an SVM with standard radial basis function kernel. It was trained on human and mouse coding and non-coding sequences and reported 95.4% and 94.2% accuracy, respectively, as well as detection accuracy rates of 96.1% and 94.7% on human test dataset comprising lncRNAs and mRNAs. A collection of known de novo lncRNA transcripts was categorized with more than 97% accuracy.

**Coding-Non-Coding Index** or **CNCI** [49] is an annotation-free classification tool for cross-species transcriptomes relying upon sequence composition. The tool uses SVMs with a standard radial basis function kernel trained on five features. Core feature is the coding domain sequence (CDS) identified by employing a system to analyze nucleotide triplets (3-mers). A 64 × 64 frequency matrix of all possible directly adjacent 3-mers is created and a sliding window of 150 nt is used to traverse each transcript and generate the six potential reading frames. From the latter a CDS most suitable to be transcribed using maximum interval sum function is calculated. The length and the quality of the most suitable CDS (*S*-score) are the two main features. CNCI achieved around 97% accuracy on 10-fold CV when trained on human protein-coding and long non-coding sequences.

**Coding-Non-Coding Identifying Tool**, or **CNIT** [50], is the successor to CNCI. It uses the same set of features and is also based on an SVM, in particular on the XGBoost implementation [51]. The model was trained on both human and *Arabidopsis thaliana* protein-coding and non-coding transcripts and tested on 10 animal and 26 plant species. On human test sequences, it achieved 98% accuracy levels.

**Coding Potential Assessment Tool**, or **CPAT** [23] was published around the same time as CNCI. It is an alignment-free tool based on a logistic regression (LR) model. Main features are ORF size, ORF coverage, Fickett score and hexamer usage bias. Training CPAT on 10,000 human coding and 10,000 non-coding transcripts taken from GENCODE and RefSeq achieved 99% accuracy on 10-fold CV. The tool was tested on an unseen dataset with 96% and 97% of sensitivity and specificity, respectively.

**LncRNApred** [52] uses a random forest (RF) model. The authors retrieved 2000 human mRNA and lncRNA transcripts each and used *self-organizing feature maps* or SOM clustering to select representative samples for training. An initial set of 89 features was reduced to 30 after feature selection. A unique feature in this approach is signal-to-noise ratio which converts a sequence into four binary sequences encoding nucleotide occurences. For every binary sequence, a complex sequence is created through *Discrete Fourier Transform* and combining all four a power spectrum is calculated. As a profound nucleotide usage bias is observed in coding transcripts [52], the power spectrum at 1/3 length of every sequence was incurred as an important feature by the authors, allowing a clear distinction between coding and lncRNA transcripts. Other features are *k*-mer features (*k* ranging from 1 to 3), GC content and sequence length. The RF model trained on these features achieved  93% CV accuracy rates for human datasets.

**FlExible Extraction of LncRNAs**, or **FEELnc** [53] is another alignment-free tool to separate lncRNAs from mRNAs based on a RF model. It computes ORFs and annotates them in five different categories: from a *strict* mode, where the ORF contains both the start and stop codon, to a *relaxed* mode, where the whole sequence is considered. *k*-mer frequencies (*k* ranging from 1 to 12) for each mRNA and lncRNA sequence are calculated and a score is assigned to each transcript per *k*-mer size. Other features used include ORF length, ORF coverage and sequence length. Human and mouse protein-coding and long non-coding transcripts were used to train the RF classifier with a reported accuracy of 91%. In addition, a number of transcripts from the NONCODE [54] data base were included to also represent species of non-model organism status. Furthermore, FEELnc applies a unique strategy for the generation of pseudo non-coding sequences in the absence of a real training set for a given species. In this case, sequences of coding transcripts are shuffled on the nucleotide level to disrupt coding signals and thus generate artificial non-coding sequences with identical nucleotide composition. This, of course, can only simulate coding feature lacking sequences and does not generate knowledge about species-specific lncRNAs.

**lncRNAnet** [55] uses a deep learning approach. The authors implemented recurrent neural networks (RNN) to determine intrinsic features of lncRNAs and convolutional neural networks (CNN) [56] to identify stop codons in ORFs. A CNN is applied to detect the region between two stop codons, as ORF detection can be difficult if start codons are non-canonical. On identification of all the stop codons, an ORF indicator is populated with values 0 and 1 for each nucleotide’s presence in an ORF, with the aim to find the longest ORF. All sequences smaller than the largest sequence are padded to match the maximum length and encoded as four-dimensional tensors. Data was taken from GENCODE v.25. Final features include the ORF indicator, ORF length and ORF coverage, which achieved 99% accuracy based on 5-fold CV. The authors emphasize that ORF indicator is a key feature in lncRNAnet, which also enabled them to successfully identify lncRNAs independent of their sequence lengths.

**lncADeep** [57] is another deep learning approach, whose declared purpose is to identify novel lncRNAs and functionally annotate them. The tool deals with both (i) full-length and (ii) partial mRNA transcripts and compares them with lncRNA transcripts. The features incorporated are ORF length, ORF coverage, entropy density profiles (EDP) [58] of ORFs, hexamer score, UTR coverage, GC content, Fickett score, HMMER [59] index, and longest CDS. The authors implemented a deep belief network (DBN) from restricted Boltzmann machines (RBM) for identification of lncRNAs. lncADeep also predicts lncRNA-protein interactions to identify lncRNA functions. Structural features like folding energy and hydrogen bonding are used besides sequence features to construct a deep neural network (DNN) to predict interactions. The tool achieved 98% sensitivity on 10-fold CV when identifying lncRNA transcripts on a training set based on full-length mRNA transcripts.

**LGC** [60] attempts to create a species-independent and coding potential-based lncRNA identification based on feature relationships between GC content and open reading frame (ORF) properties. The authors claim that the interplay between these properties shows strict divergence between coding and long non-coding RNA across all investigated species. LGC implements a maximum-likelihood approach for coding potential estimation. The GC content of sequences with 100 different levels of GC was calculated and, based on this, the probability of a stop codon present in an otherwise random sequence was inferred for each of them. An overall relationship between GC content and a stable and long ORF was approximated based on this pool. The actual classification of a sequence is then achieved by calculating a log likelihood using its GC content and the estimated probability to find a stop codon in either a coding or non-coding sequence based on the estimations above. Expected ORF lengths were calculated based on 38,811 protein-coding and 27,669 lncRNA transcripts from human. The authors report an accuracy of 95% on human data (98% with cross validation), 94% in mouse and nematode and less in fly and zebra fish.

**PLIT** [61] is an iterative RF-based tool developed in response to the discovery that a mere coding potential analysis is often insufficient to identify non-coding elements in plant transcriptomics data. It implements a feature selection method based on $L_{1}$ regularization choosing from a set of 73 features. ORF-based features were maximum ORF length, ORF coverage and mean ORF coverage. On the sequence level length, GC content, hexamer and Ficket score were taken into consideration. Overall codon bias was represented by optimal codon frequency, codon usage bias, relative codon bias, synonymous codon usage frequency, relative synonymous codon usage and weighted sum of relative entropy. The training set consisted of 22,471 coding and non-coding sequences taken from plant RNA-seq datasets. An AUC score of 0.93 is reported on a test set with a size of 25% of the training set containing an identical composition of species.

**lncRScan-SVM** [62] is another SVM based tool. It mainly depends on transcript length, exon count of a gene and mean exon length, mean conservation score, likelihood of a codon sequence in a sequence of nucleotides utilizing *txCdsPredict* from *UCSC Table Browser*, and the standard deviation of stop codon counts between three translated frames. The final feature was chosen on the basis of standard deviation of stop codon counts of lncRNA transcripts in comparison to protein-coding transcripts. It was formulated as:

$$SCS=\sqrt{\frac{1}{3}\sum_{i=0}^{2}{\left(SCC_{i}-\bar{x}\right)}^{2}},$$

where $\bar{x}=\frac{1}{3}\sum_{i=0}^{2}SCC_{i}$ is the mean stop codon count of the reading frames.

Training the model on human transcripts achieved 91% accuracy. However, a GFF format annotation file is needed together with the input sequences to set a baseline for the stop codon metric.

### 3.3. Approaches Using Potential Protein Features

**Coding Or Non-Coding**, or **CONC**, [63] is one of the earliest tools for lncRNA annotation and regularly used as a benchmark for successively developed tools. It focuses on the separation of subtypes of long non-coding from protein-coding transcripts and applies an SVM approach. The selected features consist of peptide length (four features), amino acid composition (20 features), predicted protein secondary structure content (three features), mean hydrophobicity (one feature), percentage of residues exposed to solvent (one feature) sequence compositional entropy (one feature), number of homologues (one feature) and alignment entropy (one feature). The authors also computed *k*-mers with *k* from 1 to 3 and had a final feature set consisting of 180 features. The model achieved  97% and  95% accuracy for protein-coding and non-coding sequences, respectively, on 10-fold CV.

**Coding Potential Calculator**, known as **CPC** [25] implements an SVM based classifier around six ORF-related features including ORF quality and coverage. If the ORF contains a start and a stop codon, ORF integrity is also calculated. The authors argue that protein-coding transcripts are likely to have more interactions with proteins as opposed to non-coding sequences, so number of interactions would be an important feature. They developed a method to calculate the integrity of protein-transcript interactions, assuming that a higher integrity implies a higher probability for the transcript to be coding. To compute those features, they applied the tool *framefinder* [64]. For every frame, the number of hits against known proteins is calculated via a BLASTX [65] search on the protein database UniProt Reference Clusters [66]. Hit quality is another feature considered, assuming coding transcripts to have higher quality hits. The concentration of hits among the three frames used by *framefinder* is the third feature utilized. The argument here is that coding transcripts have the hits concentrated in a single frame in contrast to non-coding transcripts. An SVM-based model was trained using a standard radial basis function kernel. CPC reported an accuracy of  96% based on 10-fold CV tested on two datasets containing non-coding transcripts and one containing protein-coding transcripts. On lncRNAs particularly, accuracy levels were reported to be around 76%. Transcripts were taken from NONCODE, Rfam [67] and EMBL data bases.

**CPC2** tool [68] is the successor of **CPC**. It is also based on an SVM but uses four features as opposed to six in the original tool. Implementing a RF model with recursive feature elimination technique on a collection of sequence intrinsic features the authors have derived Fickett score, ORF length, ORF integrity (presence of both start and stop codons in an ORF) and isoelectric point of a predicted peptide as the four main features to be fed into the SVM for classification. CPC2 was reported to not only be faster but also more accurate than its predecessor. It is also more consistent in classification accuracy rates than CPC. An accuracy of 94% was reported and 69 organisms were represented in the data sets derived from RefSeq, SwissProt [69] and Ensembl data bases.

**PORTRAIT** [70] is another SVM based tool for the separation of coding and non-coding sequences in less well-studied transcriptomes. Features are composed of purely sequence composition based properties and ORF based ones including predicted protein properties. They include nucleotide frequencies, transcript length, predicted amino acid composition, ORF length, isoelectric point, hydropathy and compositional entropy. Almost the full 2009 Rfam database alongside NONCODE and RNAdb was used in training. Sequences were split by presence or absence of an ORF and used separately to train two different classifiers, that are denoted as protein-dependent and independent. The authors report an accuracy of up to 91.9% on a case study data set.

**LncRNA-ID** [71] is another RF-based identification tool designed to identify lncRNA sequences among a set of protein-coding and long non-coding sequences. The tool employs 11 features consisting of ORF-related features, ribosomal interaction-related features and protein conservation scores using a profile hidden Markov model-based alignment. Ribosomal interaction features were built around the Kozak motif: GCCRCCAUGG, as nearly all ribosomes interact with AUG [72,73]. The ribosomal coverage was calculated as the change in binding energy. It was expected that coding transcripts had more ribosomal coverage. The accuracy reached 96% when the RF classifier was trained on these features on a human transcripts dataset.

**PlncPro** [74] is a tool for the identification of long non-coding RNA transcripts in plants using a combination of sequence-extracted features and properties of potential proteins. It uses a RF model on a total of 71 features. The latter were selected based on previous knowledge regarding properties of coding and non-coding transcripts. Primary features were length and coverage of the most probable ORFs as predicted with Framefinder [64]. Translated amino acid sequences predicted from these ORFs were queried against the SWISS-PROT database with BLASTX, extracting number of hits, significance score, total bit score and frame entropy as protein-related features. Sixty-four features based on 3-mer frequencies complete the feature set. The RF classifier was trained on coding and non-coding transcripts from the CANTATAdb.

**CREMA** [75] is a tool for ncRNA detection in plants built on stochastic gradient boosting and RFs. Training data were derived from empirically confirmed non-coding sequences in different plant species. Feature candidates for selection were mRNA length, ORF length, GC content, Ficket score, hexamer score, SwissProt alignment identity, SwissProt alignment length, mRNA length to alignment length ratio, proportion of alignment length to ORF length, transposable element presence and sequence divergence from transposable element. Test and training sets were limited to the organisms human, mouse, rice and *Arabidopsis thaliana*. The training set contained 436 confirmed lncRNA sequences, each of them extracted from lncRNAdb and a larger number of coding sequences from each species as negative set.

### 3.4. Tools Utilizing RNA Secondary Structure Properties as Features

**COME** [76] uses secondary structure conservation among other features to predict coding potential of sequences and for the identification of lncRNA transcripts. Transcripts are decomposed into subsequences (bins) and indexed. Applied features are GC content, DNA conservation, protein conservation, polyA-expression, small RNA expression, RNA secondary structure conservation, H3K36me3 and H3K4me3 modification. It should be noted that the given feature selection makes external information (i.e., the presence of histone markers and expression data) a necessity. The class probability of the mRNA label is interpreted as the overall coding potential score of a given transcript. In terms of coding potential estimation, COME scores similarly or higher than tools like CPAT, CNCI or RNAcode. The tool was trained and tested on annotated transcript data from human, mouse, nematode, *D. melanogaster* and *Arabidopsis*. Regarding the five species listed above the authors report a consistently high prediction accuracy between 92% and 97%.

**LncFinder** [20] is a tool with declared focus on novel lncRNA prediction. Three different feature categories are used. Apart from sequence composition, including features to quantify hexamer usage bias, secondary structure features and physiochemical properties of the sequences are used. The minimum free energy is calculated with RNAfold [77] from the ViennaRNA package [78]. Abandoning the *k*-mer scheme implemented in some other tools, the authors propose two feature categories to quantify hexamer usage bias. Each category has three features: genomic distance to lncRNA, genomic distance to protein-coding transcript and distance ratio. Multiple models were evaluated, namely logistic regression, SVMs, RFs, ELM and deep learning. Based on 10-fold CV, SVM was selected over the methods and reported an accuracy exceeding 96% when trained on human transcripts.

**PredLnc-GFStack** [79] is another RF-based tool using feature selection from a candidate list with a genetic algorithm. Sequence intrinsic features used are stop codon count & frequency, stop codon frequency across possible reading frames, Ficket score, GC fraction and nucleotide position frequencies. ORF features considered are ORF length, longest ORF length, ORF coverage, ORF integrity, ORF frame score and entropy density profiles. Coding sequences (CDS) are evaluated by length, coverage and percentage. Transcript length, Hexamer score, CTD, signal-to-noise ratio, UTR coverage and *k*-mer (1 through 3) frequencies are also taken into account. predLnc-GFStack also considers features specific to the potential translated peptides, these are molecular weight, isolelectric point, hydropathicity and instability index. From the candidate list a set of best-performing feature sets was selected using a genetic algorithm. In the end, the 10 best performing feature sets were used for RF training and combined to an ensemble classifier. A CV accuracy between 94% and 97% is reported. Training and test sets were constructed from coding and non-coding transcripts mainly taken from GENCODE. The training set represents human and mouse sequences, while the test set additionally included *D. melanogaster*, *S. cerevisae* and *D. rerio* RNA.

An approach that does not fit in one of the previous categories is a tool targeting the functional annotation of transcripts, going beyond the mere differentiation of coding and non-coding transcripts.

**Plaidoh** [80] was originally developed to close a gap in the detection of lncRNAs associated with cancer development (Lymphoma). The initial publication therefore focuses heavily on lncRNAs with expression in B cells. Furthermore, experiments are carried out on RNA transcripts whose average expression levels were already quantified but their exact role was still unclear. **Plaidoh** works by trying to establish a Pearson correlation between the expression levels of a given candidate transcript and associated genes in physical proximity (same cell). A strong positive or negative correlation indicates that the lncRNA in question has a regulatory function in gene expression. The subcellular localization of the transcript as obtained with immunofluorescence is also taken into account to establish lncRNA–protein interaction. Collections of **Plaidoh** results are connected to establish cancer-specific correlation patterns which can then be used to distinguish malignant cells.

## 4. Mini Benchmark on Human Transcripts and Randomly Chosen Genomic Sequences

We tested our hypothesis that four of the most impactful tools, as measured by citations when compared to their contemporaries, reviewed here (see Table 2) are capable of reliably differentiating between known coding and noncoding transcripts but would be strongly biased towards classifying incomplete or random sequence as noncoding transcripts. For this purpose we create a mini-benchmark test set of 200 randomly chosen lncRNA, 200 coding transcripts, dinucleotide shuffled versions of the latter and 194 randomly chosen sequences from the human genome (hg38) and corresponding annotation based on the RefSeq database [40] v38.

Random sequences were generated from length 200 to 3000 nts, 5–10 sequences each, utilizing bedtools random. The program bedtools getfasta [81] was used to extract 200 random genomic regions and corresponding sequences (filtering 6 only containing Ns). We generated pseudo-randomized sequences by dinucleotide shuffling the coding and lncRNA sequences and selecting a set of 200 sequences from this randomized pool.

Using this test set on the benchmark tool provided by [82] in their review on lncRNA prediction, we expected a clear majority of coding RNAs to be correctly classified as such. For lncRNAs our expectations where unclear, as some of those could actually harbor coding potential, or at least features that would falsely be recognized as such. For all reviewed tools it was expected that the randomly chosen and shuffled sequences pose a challenge. Given that only a small portion of the human genome is actually protein coding and those sequences were chosen randomly from the genome, we considered it highly unlikely that they carry any coding potential, while shuffling would probably destroy the signal altogether. Thus, we expected most of the tools to assign a non-coding label to these sequences, especially as the problem is implemented as binary classification task, i.e., no category for not coding **and** not long non-coding exists.

**Figure 2:**
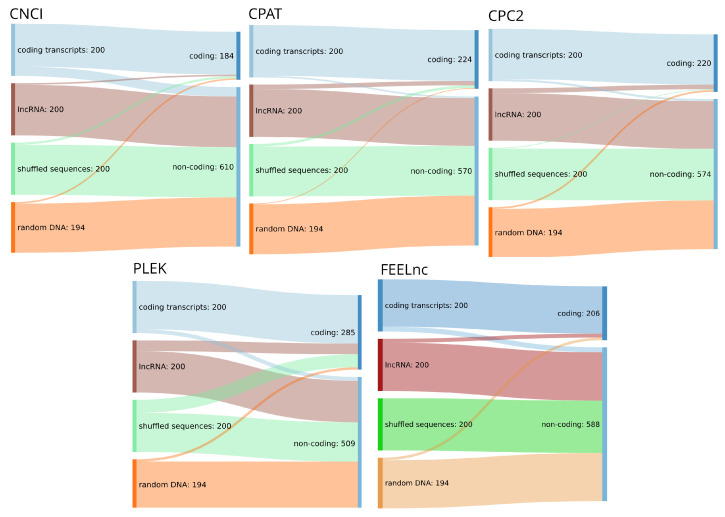
Sankey diagrams for the input dataset consisting of 200 randomly chosen lncRNA, 200 coding transcripts, dinucleotide shuffled versions of the latter and 194 randomly chosen sequences from the human genome (hg38) and corresponding annotation (RefSeq database [40] v38) and their assignment to coding or non-coding classes by five high-impact classification tools. All tools were run with standard settings where applicable.

We found that the four tools benchmarked here are in fact capable of reliably identifying confirmed coding and lncRNA transcripts (see Table 3). However, as can be seen in Figure 2, the overwhelming majority of randomized input sequences from both the shuffled and the random genomic test set were classified as non-coding transcripts. This is, by itself, not surprising as the tools discussed are typically advertised as distinguishing coding from non-coding sequences in transcripts that have already been confirmed as being expressed. However, this further supports our conclusion that although tools for the reliable classification of confirmed transcripts have existed for many years, the same tools make no progress to differentiate between true lncRNA transcripts and random DNA sequences. Therefore, we conclude that these approaches are better equipped to separate coding sequences from an ambiguous background than lncRNAs from other sequences, which would, however, be the actual task at hand. It must of course be noted, that in a typical lncRNA annotation pipeline, the first step will be the identification of transcribed sequences for the genome of interest employing for example Trinity [83] and StringTie [84], which are then classified. This approach, however, is not always feasible and assumes a level of completeness of the RNA-seq data that is rarely available. First, compared with raw genomic data, high-quality RNA-seq data are not always readily available for less well studied organisms, are sometimes difficult to acquire (e.g., compared to DNA from museum samples), and in any case requiring additional wet lab work. Second, and maybe more importantly, transcript reconstruction even from high-coverage RNA-seq data sets is by far less than perfect (see, e.g., [85] for benchmark data), and tends to produce fragmented and incomplete transcripts in particular for low coverage—which is the norm for most lncRNAs. With annotation quality rising in well-known model organisms, these less well studied species are precisely the target for mostly computational annotation efforts. For this reason, we think that the development of strategies for the prediction of non-coding and coding transcripts from a genomic background will be of utmost importance to identify promising targets in silico before conducting verification experiments. A framework that made promising progress in this direction is the RNAz [32] tool for the identification of structurally conserved regions in full-genome screens. RNAz and similar approaches, however, suffer from the disadvantage of being limited to classifying alignments instead of sequences and being blind to non-coding RNA transcripts that show little or no structural conservation. Again, many ncRNAs fall into this group. For these reasons, there is a pressing need for the development of genome based in silico prediction pipelines for lncRNA loci building upon the approaches outlined here.

The non-binary classification problem outlined here could of course be partially circumvented by using a tool that not only supplies a predicted class label but also a probability or coding potential score to quantify the likelihood of a given transcript being in a certain class. Such tools include, among others, FEELnc and CPC, both of which yield a coding potential score. Based on this, a third, undecided category with ambiguous coding potential could be introduced. However, here we encounter the problem that the exact cutoffs of these classes are not identical and not easily defined for each species. Therefore, while promising, this approach goes beyond the scope of this mini-benchmark.

## 5. Discussion

The large number of published tools for the detection and classification of lncRNAs and the diversity of the underlying approaches emphasizes the continuing interest in and the practical importance of this particular classification problem. In the previous section we reviewed 22 dedicated tools, selected for their relative impact in this field of research (see Table 1). The methods utilize a wide variety of different properties and features of the nucleotide sequence, the predicted translation product(s), and in some case also of the transcript’s RNA secondary structure. They also employ a diverse portfolio of distinct machine learning algorithms. Most methods utilize features that directly assess the coding potential, i.e., the likelihood of a given sequence to encode a protein.

A core issue is that current annotation pipelines are typically dependent on accurate transcriptome annotation data for training, rendering de novo annotation of newly assembled genomes impossible unless a very closely related genome with accurate annotation is already available and can be used for training. Furthermore, the quality of transcriptome annotation, thus, has a direct impact on the prediction accuracy and the former is rarely complete. Protein-coding transcripts can be full-length as well as partial-length. Together with incomplete splicing information this directly impacts exon annotation, the latter being a key part of training sets as units of protein coding potential are usually incomplete. The same holds true for annotated lncRNAs, used as positive set for training.

An alternative option, as discussed in Section 3, lies in the artificial generation of data from the other half of the training set, namely in the creation of non-coding sequences from coding ones by shuffling. While a valid approach in principle, it has to be applied with much caution in practice. Any shuffling procedure explicitly or implicitly makes the assumption that certain features are preserved, while everything else is perfectly randomized in the background. For example, a shuffling process will necessarily disrupt RNA secondary structure signals by reordering potential base pairs unless specifically designed to conserve a specific structure. This makes the shuffling approach less useful for tools considering RNA secondary structure, such as COME, and potential protein properties implied in the sequence, such as CONC and CPC. However, shuffling may be a viable training set generation approach for tools primarily using *k*-mer frequencies and other purely sequence-intrinsic features. Still, a shuffled coding sequence does not necessarily mirror *k*-mer frequencies and other sequence biases typically encountered in genuine lncRNA transcripts. Shuffling procedures thus are safely applicable to the generation of randomized background data when distinguishing features are known, such as codon patterns versus retained (di)nucleotide frequencies for the purpose of coding sequence detection. They are highly problematic, however, in cases where it is not clear at the outset which features separate classes and thus have to be randomized by shuffling in contrast to features common to the classes, which therefore must be preserved by the shuffling procedure, such as dinucleotide frequencies in a comparison of lncRNA exons vs. UTRs or intronic sequences.

### 5.1. Classification Accuracy in the Context of Used Features

The majority of tools presented here report accuracy metrics in the high 90% interval on their respective validation sets (Table 1). This is consistent with observations in our benchmark (see Section 4). However, no clear link can be established between commonly used features and specific machine learning algorithms. In fact, some of the most accurate tools utilize little more than variations of *k*-mer frequencies or other metrics for sequence composition on a lexicographic level. This is further backed by publications explicitly selecting the most significant features from a larger pool of candidates for classification purposes, i.e., COME and DeepLnc. In our interpretation, no clear correlation can be drawn between the use of more sophisticated metrics and features, for example, physical properties of hypothetical encoded proteins, and a higher rate of classification success. In fact, assuming that authors do not underreport the reliability of used features, it seems hard to justify the use of more complicated approaches for many use cases. This is especially worth considering as the physico-chemical properties of putative proteins in the previous example are typically estimated based on the in silico translated amino acid chain [23], which in turn is therefore already implicitly contained in the input sequence. If the nucleotide sequence is evolved to encode a protein with specific properties and there is a qualitative difference to one that is not, it would be reasonable to assume that this effect is already detectable on the level of the nucleotide sequence. Given the above considerations, there is currently little evidence that the introduction of new features in comparison with long-established coding potential metrics such as the Ficket- and Hexamer-scores yields significantly more information, as long as functional annotation of a predicted lncRNA is not considered.

Furthermore, it has to be noted that the direct comparison of accuracy scores between different classification tools is difficult, especially if they were designed for different use cases and built around incomparable datasets. The same holds true for the comparison of the discriminative power of individual features. One particular problem are the empirical differences in dinucleotide composition, frequency of structural and sequence motifs between species and different realms of life [86,87,88]. For this reason, individual differences in discriminative power may be a result of subtle differences in either training set or a qualitative difference between features for different applications. All this makes a fair comparison of classifier quality on a case-by-case basis exceedingly hard. To our knowledge, there is no gold standard benchmark set for the estimation of lncRNA identification capabilities, especially one covering all the aspects discussed here. Ideally, such a benchmark set would not only consist of coding and non-coding sequences but also contain random and incomplete sequences. Furthermore, although only some tools are applied in a cross-species fashion, this would also need to be part of such a gold standard. We conclude that establishing such a benchmark dataset is a complex research endeavor, however, one we deem worthwhile to engage in as it would have a direct and strong impact on the field of lncRNA research.

### 5.2. Comparing Performance of lncRNA and Coding Potential Classifiers

Direct comparison of a numeric accuracy score is probably not the most effective way to estimate the overall impact of an approach on the field of functional gene annotation, especially since a gold-standard benchmark dataset is not available. We therefore analyze (a) the involvement of a given approach in de novo identifications of non-coding and functional transcripts and (b) the overall methodological impact on the development and refinement of existing classification approaches. We reviewed all tools discussed here for these criteria and identified five key contributors that had a significant impact on this field of research (see Table 2). Of these tools, CPC has by far the strongest impact as a whole, having been cited more than 1800 times (as of November 2021). The tool has been involved in the discovery of, among many others, the BANCR [89] and pnky [90] lncRNAs and has been cited numerous times in articles reviewing the state of the art in functional RNA annotation (compare [91,92,93]). Furthermore, it has been named as a methodological source in the field of coding potential estimation by a majority of the tools reviewed here (i.e., [18,23,68]).

The direct successor of CPC, CPC2, was developed as a methodological improvement of CPC and has as such also been a contributor to the identification and annotation of multiple lncRNA transcripts, e.g., MNX1-AS1 [94] and DCST1-AS1 [95], among others. As such we consider it likely that it will follow CPC as one of the most cited tools used for coding potential estimation and target identification of potential new lncRNAs in transcriptome screens.

CNCI has also contributed significantly to the development of coding-potential-based assessment methods, applying an approach based on identifying the most probable coding sequence in a given transcript. The ability to screen for coding sections in a sequence while considering all possible reading frames has especially led to the discovery of small peptides encoded in long functional RNA sequences previously thought to be fully non-coding. It has also been used for the investigation of lncRNA features in plant transcripts [96]. Another contribution partially attributed to the application of CNCI has been the discovery of the ZEB1-AS1 lncRNA [97]. CNCI is one of few tools discussed here that is explicitly trained on plant transcripts in particular, which can be considered an advantage for future investigations, as the annotation of functional RNAs in plants is currently highly incomplete. It is also notable that CNCI, just as CPC and CPC2, uses a comparably small number of features that are all primarily based on sequence composition.

Another standard tool assessing coding potential often cited is CPAT. It has, among others, been involved in the annotation of CCAT2 [98] and is, alongside CPC, often used for the purpose of performance comparison and benchmark of other methods (e.g., [62,68,71] and others). Remarkably, it seems to be responsible for the reintroduction of the Ficket- and hexamer-score features, two features which have already been described as very strong discriminators of coding sequences in the early stages of gene annotation (compare [22,24]). PLEK, basing its assessment of a transcript exclusively around *k*-mer frequencies, is also on the list of high-impact lncRNA identification tools. It contributed significantly to genome-wide annotation of lncRNA associated with chlorantraniliprole resistance in *Plutella xylostella* together with CPAT and CPC [99]. This is of specific interest as PLEK was originally trained on vertebrate species, but yields sufficient accuracy on some non-vertebrate species as well.

The five tools discussed here assess coding potential based on sequence-intrinsic features and open reading frame properties, with the exception of CPC2, which also takes physico-chemical properties of predicted peptides into consideration. Of note in particular is PLEK, which outperforms many other tools in terms of reported accuracy and has seen considerable success in real-life annotation projects, demonstrating that its minimalistic set of features is sufficient for a reliable estimation of coding potential. Given the successes of these approaches, it can be concluded that for the de novo detection of lncRNA transcripts, the assessment of coding potential based on sequence intrinsic features is both possible and efficient, as already discussed by others [100]. Furthermore, the underlying mechanisms seem to be well-enough understood and flexible that even features used in approaches published more than a decade ago (i.e., those in CPC) still see widespread and successful usage in contemporary research. Based on this, we estimate that the approaches discussed here will probably lead to further discovery of new lncRNA transcripts in the next years. However, identification and annotation of lncRNA genes in the genome can only be the first step. Based on a continuously improved annotation base, the next goal has to be the precise classification of function and biological mechanisms encountered in lncRNAs.

### 5.3. Functional Classification of lncRNA

An emerging field of study is the identification and categorization of lncRNA by biological mechanism and function, e.g., binding of other macromolecules or chromatin remodeling [101,102]. At the moment there is only a small number of lncRNAs functionally annotated, most of them in the field of cancer research (compare [5,6,103]). A vexing question is the applicability of the features presented here to the problem of functional categorization. With the exception of lncADeep and Plaidoh, however, no tool reviewed here is intended for functional annotation. Currently, more than 20,000 genes involved in the transcription of lncRNAs have been annotated in human, more being added each year. However, the amount of available lncRNA examples with known biological function and purpose is only a very small subset. This problem is made more complex by the varying reliability of these annotations. Therefore, it is currently difficult to conduct a study with sufficient statistical power. We believe however, that given a sufficiently large set of lncRNAs with well-understood mechanism, a systematic investigation of correlation between function and features discussed here is worthwhile. This is especially true as lncRNAs are known to be involved in a wide range of pathophysiological processes and are subject to intensive research in new therapeutic approaches (compare [104,105,106,107]). A better understanding and classification of involved mechanisms could thus give rise to potential refined diagnostic methods downstream.

## 6. Concluding Remarks

The classification task of distinguishing lncRNAs from protein coding mRNAs does not seem to require the inclusion of extensive numbers of different features or of sophisticated features describing the putative translation product. At least the evaluation of the literature on the tools studied here does not suggest a systematic, beneficial influence of such features on the classification accuracy.

The methods discussed here classify any sufficiently long transcript with low enough coding potential as a lncRNA. This is further emphasized by our mini-benchmark in Section 4. Although they are advertized and evaluated for the purpose of lncRNA identification, most tools should rather be viewed as *coding sequence classifiers*. These two tasks are equivalent only if the input is guaranteed to be a complete transcript. The tools reviewed here make this assumption, albeit often tacitly. In a more general setting, i.e., when presented with an arbitrary piece of genomic sequence or a fragment of a transcript, the tasks differ. A **lncRNA detector** would be expected to distinguish whether the input sequence is part of a (processed or unspliced) lncRNA as opposed to, say, an intronic or untranscribed part of the genome or the UTR of a coding mRNA. None of the tools reviewed here were designed or trained for this task. In practice, the available tools therefore can only be expected to distinguish between an input sequence that contains a protein-coding subsequence, and those that do not. Figure 2 shows that the most commonly used tools indeed classify lncRNAs together with shuffled sequences and randomly picked genomic DNA in their “non-coding” category.

Due to comparably low expression levels, lncRNA transcripts often cannot be assembled to yield the complete sequence. By construction, the tools reviewed above must be expected to classify a large piece of the untranslated regions (UTRs) of a coding mRNA as “lncRNAs”. This may lead (or already has led) to an overprediction of lncRNAs in genome annotations. Classifiers that can distinguish parts of lncRNAs from UTRs thus would be of immediate practical use. So far, this—presumably much harder—task has received very little attention. With the exception of PLIT, none of the reviewed tools claim to be able to handle input directly from RNA-Seq experiments, highlighting the need for novel algorithms to enable lncRNA detection from the vast amount of publicly available datasets. Furthermore, we have shown high numbers of false non-coding transcript classifications for a small test set of randomly chosen coding, non-coding, nucleotide-shuffled and random genomic sequences of length 200–3000 nt extracted from the human genome (see Section 4). This emphasizes the need for a true **lncRNA detector** even more.

The assumption of complete input transcripts also impacts test and training sets. Since all tools rely on available transcript annotation, training results may depend on the completeness in particular of the non-coding transcripts. This is the case when features such as poly-A sites are used, which also exist on at least a subset of non-coding transcripts. It is conceivable that the classification is influenced by a confounding effect that associates complete transcripts preferentially with coding mRNAs and incomplete transcripts with lncRNAs. To our knowledge, such potential biases have not been investigated systematically so far. While third-generation sequencing methods have the potential to alleviate the potential bias through incomplete sequences, it is unlikely that in particular lowly expressed transcripts will be sequenced comprehensively by NanoPore or PacBio in the near future.

A question that also remains largely unanswered is to what extent clade-specific differences affect classification. This is likely not a big issue as long as the task is only the separation of coding from non-coding sequence, but plays a role when more sophisticated tasks need to be addressed.

The emerging big challenge in the study of lncRNAs will not only be the annotation, but also the categorization by molecular mechanisms and biological function in subgroups. Unsuperivsed clustering based on *k*-mer distances showed that there is some correlation of lncRNA function and sequence features [19]. It remains an open question, however, if there are distinct classes of lncRNAs with different biological functions and/or molecular mechanisms, or whether the space of lncRNA functions and mechanisms rather form a densely populated continuum. A recent study of miRNA and snoRNA host genes suggests that the relationships between features, functions and mechanisms is likely far from simple [108]. It will likely be the case that typical lncRNAs are involved in multiple functions and take part in multiple molecular mechanisms. The associations of function, mechanism and sequence, therefore, might not be well modelled by simple classification tasks.   

## Figures and Tables

**Table 1:** LncRNA detection tools: Some of the essential characteristics of the reviewed lncRNA detection tools are summarized. Latter were ordered by year of release. Furthermore, the average number of citations accumulated per year is given as an approximate impact metric. To minimize redundancy, only tools that were on average cited more than 10 times per year were included in this review, unless they contributed an explicitly unique approach in their feature design not encountered elsewhere, i.e., BASiNet and PLIT.

Tool	Year	Algorithm	Species	Features	Performance	Mean Citations per Year
**CONC**	2006	SVM	Eukaryotes (both protein-coding and non-coding genes)	peptide length, amino acid composition, predicted secondary structure content, mean hydrophobicity, percentage of residues exposed to solvent, sequence compositional entropy, number of homologues, alignment entropy	10-fold CV on protein-coding: F1-score: 97.4% ☼ Precision: 97.1% ☼ Recall: 97.8% ◙ On non-coding: F1-score: 94.5% ☼ Precision: 95.2% ☼ Recall: 93.8%	12.4
**CPC**	2007	SVM	Eukaryotes (both protein-coding and non-coding genes)	ORF features (quality, coverage, integrity), number of BLASTX hits, hit score, frame score	10-fold CV: 95.77% ☼ Accuracy on Rfam database (non-coding): 98.62% ☼ RNADB (non-coding): 91.5% ☼ EMBL cds (protein-coding): 99.08% ◙ Accuracy in lncRNA detection: 76.2%	131.8
**PORTRAIT**	2009	SVM	Species neutral, case study on *Paracoccidioides brasiliensis* and 5 other fungi	ORF length, isoelectric point, hydropathy, compositional entropy	Accuracy: 91.9% ☼ Specificity: 95% ☼ Sensitivity: 86.4% ☼	10.2
**CNCI**	2013	SVM	Vertebrates, plants, orangutan	adjacent nucleotide triplets, sequence score, codon-bias, most-like CDS (MLCDS), length-percentage, score-distance	10-fold CV accuracy on human: 97.3% ◙ Minimum average error for vertebrates < 0.1 ☼ Plants: 0.24	111.3
**CPAT**	2013	Logistic regression	human	ORF length, ORF to transcript length ratio, Fickett score, hexamer usage bias	10-fold accuracy: 99% ☼ Precision: 96%	135.6
**iSeeRNA**	2013	SVM	human, mouse	frequency of six *k*-mers (GC, CT, TAG, TGT, ACG, TCG), conservation score, ORF length and proportion	Accuracy in human lncRNA detection: 96.1% ☼ Mouse: 94.2% ◙ Accuracy in human protein-coding gene detection: 94.7% ☼ Mouse: 92.7%	19.5
**PLEK**	2014	SVM	11 vertebrates	*k*-mer frequency (for *k* = [1,5])	10-fold CV accuracy: 95.6%	50.5
**lncRScan-SVM**	2015	SVM	human, mouse	sum of lengths of exons, frequency of exons, mean exon length, standard deviation of stop codon frequency, txCdsPredict	Two test sets created based on (i) random protein-coding and lncRNA sequences and (ii) only dissimilar sequences. Accuracy on set A for human: 91.54% ☼ Mouse: 92.21% ◙ On set B for human: 91.45% ☼ Mouse: 92.2% ◙ MCC on set A for human: 83.17% ☼ Mouse: 84.59% ◙ On set B for human: 82.99% ☼ Mouse: 84.69% ◙ AUC on set A for human: 96.39% ☼ Mouse: 96.62% ◙ On set B for human: 96.39% ☼ Mouse: 96.64% ◙	13.2
**LncRNA-ID**	2015	Random forests	human, mouse	ORF related features, ribosomal interaction related features, protein conservation scores	Specificity on human: 95.28% ☼ Mouse: 92.1% ◙ Recall on human: 96.28% ☼ Mouse: 94.45% ◙ Accuracy on human: 95.78% ☼ Mouse: 93.28%	12.7
**COME**	2016	Random forest	human, mouse, nematode, fruit fly, arabidopsis	GC content, DNA sequence conservation, protein conservation, polyA abundance, RNA secondary structure conservation, ORF score, expression specificity score	Accuracy: human (93.7%), arabidopsis (98.3%), mouse (89.8%), nematode (98.9%), fruit fly (98.4%)	16.2
**DeepLNC**	2016	Deep neural network	human	*k*-mer combinations (for *k* = [2,5])	10-fold CV accuracy: 98.07% ☼ MCC: 96% ☼ Recall: 98.98% ☼ Precision: 97.14% ☼ AUC: 99.3%	12.4
**FEELnc**	2017	Random forests	human, mouse	ORF features (coverage, length), sequence length, coding potential score, *k*-mer score based on frequency	Accuracy for human: 91.9% ☼ Mouse: 93.9% ◙ Sensitivity for human: 92.3% ☼ Mouse: 93.8% ◙ Specificity for human: 91.5% ☼ Mouse: 94.1% ◙ F score for human: 91.9% ☼ Mouse: 95.6% ◙ MCC for human: 83.8% ☼ Mouse: 85.6%	49.5
**CPC2**	2017	Random forest	Species neutral, trained and tested on animals and plants (both protein-coding and non-coding genes)	ORF features (quality, coverage, integrity), Fickett score, isoelectric point	Accuracy: 96.1% ☼ Specificity: 97% ☼ Recall: 95.2% ◙ Accuracy in lncRNA detection: 94.2%	97.3
**PlncPRO**	2017	Random forest	plants	64 *k*-mer frequencies, ORF coverage, ORF score, BLASTX: hits, significance, total bit score, frame entropy		13.8
**lncRNAnet**	2018	Convolutional neural network, recurrent neural network	human, mouse	sequence, ORF features (length, coverage, indicator)	5-fold accuracy: 99% ◙ Accuracy on human: 91.79% ☼ Mouse: 91.83% ◙ Specificity on human: 87.66% ☼ Mouse: 89.03% ◙ Sensitivity on human: 95.91% ☼ Mouse: 94.63% ◙ AUC on human: 96.72% ☼ Mouse: 96.67% *↯*Also available are test results on 11 different species and on experimental NGS data.	22
**lncADeep**	2018	Deep belief network	human, mouse	ORF features (length, coverage, hexamer score of longest ORF, entropy density profile), UTR coverage, GC content of UTRs, Fickett score, HMMER index	Precision for lncRNA detection from full-length mRNA transcripts: 97.2% ☼ Recall: 98.1% ☼ Average harmonic mean: 97.7% ◙ Precision for lncRNA detection from both full and partial-length mRNA transcripts: 94.5% ☼ Recall: 93.8% ☼ Average harmonic mean: 94.2% ◙ Precision for lncRNA detection from partial-length mRNA transcripts: 90.3% ☼ Recall: 93.8% ☼ Average harmonic mean: 92%	22.6
**LncFinder**	2018	SVM	Trained on human, tested on human, mouse, wheat, zebrafish, chicken	genomic distance to lncRNA, genomic distance to protein-coding transcript, distance ratio, EIIP value	10-fold CV accuracy: 96.87%	17.6
**BASiNET**	2018	Decision tree on complex networks	datasets from PLEK and CPC2	average shortest path, average betweenness centrality, average degree, assortativity, maximum degree, minimum degree, clustering coefficient, motif frequency		8.6
**CREMA**	2018	Random forest	human, mouse, rice, arabidopsis	length, GC content, hexamer score, alignment identity, ratio of alignment length and mRNA length, ratio of alignment length and ORF length, transposable elements, sequence divergence from transposable element, ORF length, Ficket score		11
**CNIT**	2019	SVM	11 animal species, 26 plant species	max_score of MLCDS, standard deviation of MLCDS scores and MLCDS lengths, frequency of 64 codons	Accuracy on human: 98% ☼ Mouse: 95% ☼ Zebrafish: 93% ☼ Fruit fly: 93% ☼ arabidopsis: 98%	20.5
**PLIT**	2019	Random forest	plants: arabidopsis, soy bean, rice, tomato, sorghum, vine grape, maize	transcript length, GC content, Ficket-score, hexamer score, maximum ORF length, ORF coverage, mean ORF coverage, codon bias	AUC: 93.3 % for everything except S. bicolor (75%) and arabidopsis (85%)	8.5
**LGC**	2019	Feature relationship	human, mouse, zebrafish, nematode, rice, tomato	GC content, ORF length, coding potential score	Accuracy, 10 fold cross-validation: human (94.5%), mouse (93.6%), zebrafish (88.4%), nematode (93.3%), tomato (93.3%), rice (96.3%)	12.5

Abbreviations used in the table: Mathew’s correlation coefficient (MCC) with $MCC=\frac{TP\times TN-FP\times FN}{\left(TP+FP)(TP+FN)(TN+FP)(TN+FN\right)}$, cross validation (CV), area under curve (AUC).

**Table 2:** Tools with the highest impact on lncRNA annotation research ordered by year. A tool was considered to be of high impact if it had accumulated a significantly larger number of citations in the same time frame than a competing tool and could be shown to be involved in the identification of multiple bona fide non-coding RNAs that were previously undiscovered. Number of citations refers to the last access on 11 November 2021.

Tool	Year	Algorithm	Citations	Species
CPC	2007	SVM	1857	Eukaryotes (protein-coding and non-coding transcripts)
CNCI	2013	SVM	898	Vertebrates, plants, orangutan
CPAT	2013	logistic regression	1105	human
PLEK	2014	SVM	359	11 vertebrate species
CPC2	2017	Random forest	406	Eukaryotes (protein-coding and non-coding transcripts)
FEELnc	2017	Random Forest	198	human, mouse

**Table 3:** Classification performance measures of benchmarked tools. We calculated sensitivity, specificity, precision and raw accuracy based on classification of 400 randomly chosen human transcripts, 200 of which were protein-coding and 200 were confirmed non-coding RNAs of 200 nt to 3500 nt length. The transcripts used in this benchmark were extracted from the RefSeq database [40] (ver. 38). Bold faced values highlight the maximum achieved for each measurement.

Tool	Sensitivity	Specificity	Accuracy	Precision
CNCI	0.82	**0.97**	0.895	**0.96**
CPAT	**0.97**	0.92	**0.94**	0.92
PLEK	0.93	0.79	0.86	0.82
CPC2	0.96	0.91	**0.94**	0.91
FEELnc	0.91	0.93	0.92	0.92

## Data Availability

Data are available from the authors’ web site http://www.bioinf.uni-leipzig.de/supplements/21-004.
